# Supplementary material for: Complexome profiling of the Chlamydomonas psb28 mutant reveals TEF5 as an early PSII assembly factor
Source: Plant Cell. 2025 Mar 17;37(6):koaf055. doi: 10.1093/plcell/koaf055 (PMC12164590; doi:10.1093/plcell/koaf055)
Supplement: koaf055_Supplementary_Data [file koaf055_supplementary_data.zip › psb28_interactive.zip!/plots/intensity/qualityPlots/peptides/SRR24A_Cre01.g002787.t1.1.html]

### Cre01.g002787.t1.1

Trivial name: SRR24A  
SeqCoverage [median]: 0.70  
Mass: 453.87 kDa  
  
 

| Sequence | MissedCleavage | Mass [Da] | wtIntensity | muIntensity | Score |
| --- | --- | --- | --- | --- | --- |
| CRELTLLSGLSLLR | 1 | 1572.90 | 344.75 | 0.00 | 37.95 |
| LPADPRPGTGANALTSLR | 1 | 1805.97 | 63734.59 | 38450.78 | 51.47 |
|  |  |  | 64079.3 | 38450.8 |  |
